# What Does It Take for an Organic Closed Shell Molecule to Become Magnetic?

**DOI:** 10.1021/acs.jpclett.5c03056

**Published:** 2025-11-17

**Authors:** Jonas Fransson

**Affiliations:** Department of Physics and Astronomy, Uppsala University, Box 516, 752 37 Uppsala, Sweden

## Abstract

Molecular magnetism has long been associated with metal–organic framework and organic radicals. The former offer localized d- or f-shell electrons that form a local spin moment whereas the latter comprise unpaired electrons in the outer shell. While these are natural classes of molecules to consider in this context, questions concerning magnetic properties of closed shell organic molecules interfaced with the reservoir has emerged in the wake of recent experimental surprises. In studies pertaining to the chirality induced spin selectivity effect, chiral organic closed shell molecules have been used to provide, for instance, the anomalous Hall effect and Yu–Shiba–Rusinov-like states. Both these effects require the presence of either ferromagnetism or localized spin moments. While many attempts to construct theoretical models for the chirality induced spin selectivity effects are based on uncorrelated electrons, these are bound to fail for the same reason. In this Perspective, it is argued that the chirality induced spin selectivity effect is intimately related with the emergence of stabilized spin configurations in the chiral molecule when interfaced with a reservoir.

## Graphical abstract

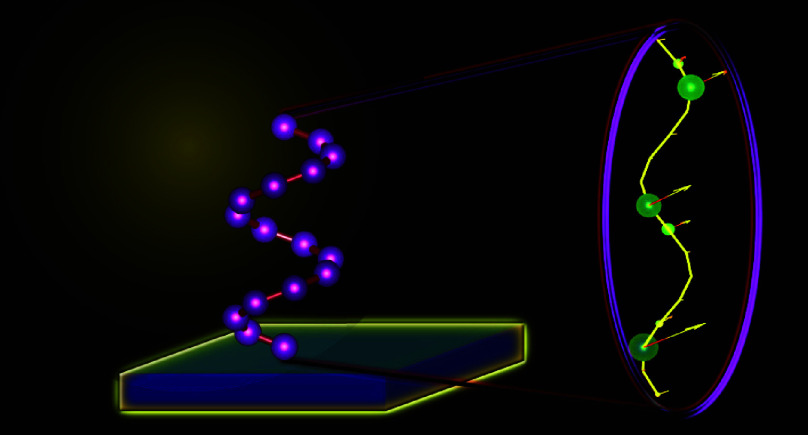

The concept of magnetic order is developed in the context of condensed matter, which may be one reason as to why magnetism is strongly associated with the solid state. And yes, when considering ordered states, one always has to keep in mind that this terminology reflects long range of a kind that is only accessible at the macroscopic scale—strictly speaking long-range order requires access to infinity to which macroscopically large objects serve as good enough approximations. Nevertheless, just because magnetism can be associated with long-range order, reflecting, e.g., ferro- and antiferromagnetism, does not mean that the terminology is not appropriate in other circumstances. It is correct to talk about magnetic states and magnetic moments in the context of molecules and structures involving molecules.

When thinking about magnetic molecules, lots of questions come to mind since it is not obvious what the term *magnetic molecule* refers to. It may be that the molecule comprises metallic ions^1–3^ which assume a net integer or half-integer spin, although there is no net magnetic moment since the environment it is embedded in leads to a superparamagnetic state of the ion. It may be because the environment that governs the spin state is either isotropic or anisotropic in such a way that the favored states are degenerate or having a projection onto zero spin. While the metallic element is in an ionic state, the molecule itself need not be a radical. Radicals are also magnetically active,^4–7^ such that electron-spin resonances of various kinds can be employed for, e.g., characterization and chemical analysis. On the other hand, by similar arguments as for the metallic ion, there is normally no net magnetic moment associated with a radical.

Organic molecules are often closed shell structures, which means that they are in a singlet state and, therefore, should have little, if anything, to do with magnetism. This is, of course, based on molecules being isolated from a surrounding with which it can exchange anything that may cause changes to its magnetic properties. A closed shell structure in a vacuum can by this perception not assume a stable spin configuration. The singlet state is, however, an entangled mixture of different spin configurations. The simplest example of a spin singlet is a dimer of spin 1/2 particles and may be expressed as |*S* = 0⟩ = (|↑⟩|↓⟩ – |↓⟩|↑⟩)/ $\sqrt{2}$ . Should one make attempts to measure the singlet state, one would likely obtain an equally weighted statistical distribution of the two configurations. One interpretation of this result would suggest that at any given time instant, the dimer is in either of the two configurations involved in the singlet while on average it spends equal time in both. In this sense, there are two reasons to why the singlet state has vanishing magnetic moment. Each of the configurations individually have zero magnetic moment and the fluctuations also lead to the magnetic moment of each constituent atom vanishing. It should be remarked, however, that while this way of viewing the entangled state may not be entirely correct, it nonetheless helps the intuition of what may take place when the molecule is adsorbed on a metal, as will be discussed below.

Because of the seemingly magnetic inactivity of organic closed shell molecules, it is remarkably surprising that chiral organic closed shell molecules nearly without exception respond anisotropically to external magnetism. And that the photoemission spectroscopy of a chiral organic closed shell molecule is spin-polarized. The phenomenon is referred to as the *chirality induced spin selectivity* effect,^8–11^ an effect that has contributed to changing our perception of what is magnetically active and what might cause this activity. What is meant here by *external magnetism* is a magnetized ferromagnet onto which the molecule is adsorbed in some way.

While magnetic molecules comprising either d- or f-electrons and molecular magnetism based on radicals are fields that have been researched a fair amount in the past two decades or so, questions related to which magnetic properties closed shell molecules may and may not demonstrate have just begun to be asked. This is for natural reasons, of course, because why would anyone think that, e.g., a DNA molecule should have anything to do with magnetism. One reason why this question is raised at all is the chirality induced spin selectivity effect.

As a concept, the chirality induced spin selectivity effect is well-established in physics, chemistry, and biology.^11^ Briefly, whenever electrons flow through a chiral material, they will likely be spin-polarized in the direction they propagate.^8^ Aside from its intrinsic interest, this phenomenon has implications, for example, for devices and for our understanding of biological electrochemistry. In aerobes, large electron currents, typically tens of amperes in the resting human, flow from metabolism to oxygen.^12^ What makes spin important in this process of respiration is that the ultimate electron acceptor, dioxygen, is a ground state triplet.

One of the reactions involved in the reduction of oxygen leading to water involves a two-electron transfer. It has been shown that this reaction is facilitated by spin polarization.^13^ At a ferromagnetic electrode, oxygen reduction proceeds faster in a magnetic field. Remarkably, general anesthetics, known to affect cellular respiration, markedly reduce spin polarization at a ferromagnetic electrode.^14^ This said, the extent and importance of spin polarization in living systems remains unknown. Direct two-terminal measurements of electron currents in biology are usually impossible.

In contrast to photoemission,^9^ which provides a clear demonstration that the emitted electrons are spin-polarized, there is only indirect evidence of charge currents flowing through chiral molecules are too.^10,15,16^ Discerning the magnetic properties of chiral structures in the absence of external magnetic forces presents clear challenges and is, therefore, missing to a large extent. Basically, the only rather clear indications that chiral molecules may acquire magnetic properties are the observations of the (anomalous) Hall effect^17,18^ and resonances within the supconducting gap of *s*-wave superconductors,^19^ so-called Yu–Shiba–Rusinov states; see Figure 1. Both these effects may be interpreted as originating from local spin moments. Should the currents be shown to be actually spin-polarized, it may amount to noninvasive methods for assessing whether the electron currents flowing in a living organism are spin-polarized. Following this thought, it was recently predicted that chiral molecules should acquire an inhomogeneous spin distribution under a charge current flux.^20^

**Figure 1:**
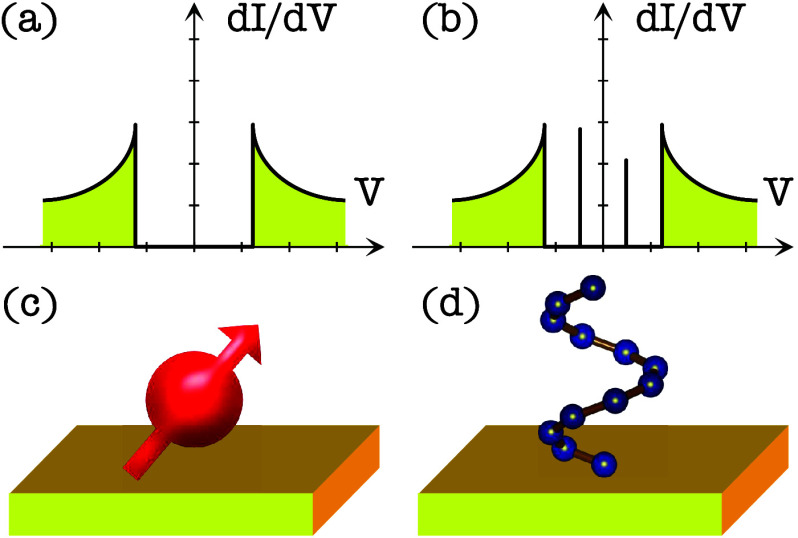
Expected differential conductance (d*I*/d*V*) plots at local probing measurements on (a) a pristine defect free superconducting surface and (b) the same surface with a magnetic defect. The magnetic defect (c) gives rise to in-gap resonances, Yu–Shiba–Rusinov states, due to scattering off the local spin moment, similar to the resonances observed in the presence of chiral molecules (d).

In this Perspective, the focus is not on the chirality induced spin selectivity effect in itself, but rather questions related to its origin, which may be associated with spontaneous formation of molecular local spin moments. And, looking further, the prospect is a revised view of the origins of magnetism and time-reversal broken states. The discussion here concerns magnetic phenomenology as an emergent effect when interfacing micro- and macro-structures with very different characteristics.

## The Effect of Interfacing with the Reservoir

While the closed shell molecule inevitably is forced into a singlet state, it remains an open question which properties it actually acquires when interfaced with a reservoir. Here, a reservoir may be a metal or semiconductor, but could also be a large macromolecule. The important aspect is that it can be thought of as a source or drain of a macroscopic number of degrees of freedom such that its properties do not change, at least approximately, in the presence of the adsorbed molecule. By the interfacing, the molecule is exposed to the macroscopic environment which may or may not significantly modify its properties depending on its internal structure.

It is important to appreciate the fact that a molecule which is isolated from its environment and a molecule adsorbed on a reservoir are not comparable objects. While the spectrum, for instance, in the isolated molecule is discrete, it is continuous in the adsorbed molecule. The meaning of this statement is that the lifetimes of the peaks in the discrete spectrum are all infinite whereas they are finite in the continuous spectrum. This seemingly innocent difference has a deeper meaning than a simple discrimination between the spectral properties. Upon adsorption the molecule becomes an open system, and as a result of this, openness is, therefore, not bound to assume the singlet state. There may be configurations that are more probable and others less. It is a matter of the molecular structure and electron correlations.

By first addressing the electron correlations, a word of caution has to be applied. In this Perspective, the term *correlation* is used with its linguistic connotation, that there is a mutual relation between two or more electrons. Physically, this means that there is an effective electron–electron interaction which may originate in direct Coulomb forces or indirectly through interactions between electrons and moving nuclei.^21^ The obvious reason for including electron correlations in molecular electronic structure calculations is to obtain the spin singlet ground state. Without electron correlations, no such state can be obtained since the noninteracting ground state is a mixture of both parallel and antiparallel spins and does not conform with the spin singlet symmetries. Consequently, the ground state for the electron dimer mentioned above is of the form |2*e* ^–^⟩ = α_1_|*↑*⟩|*↑*⟩ + α_2_|*↑*⟩|*↓*⟩ + α_3_|*↓*⟩|*↑*⟩ + α_4_|*↓*⟩|*↓*⟩, where α_*i*_, *i* = 1, 2, 3, 4, is constant. Each electron is not restricted by its environment (the other electron) and may freely assume any spin projection.

Interestingly, as the interface with the reservoir turns the molecule into an effectively open system, the interplay between the internal electron correlations and the influence of the reservoir may result in properties that neither of the subsystems possess when disconnected. Such changes in the properties are illustrated by the observations of a Hall voltage when passing a charge current through a metal covered with chiral molecules. The classical Hall effect is obtained by driving a charge current through a metal to which a magnetic field is applied perpendicular to the current. This gives rise to a charge separation in the direction perpendicular to both the current and magnetic field, called the Hall voltage. The magnetic field may be from an external source or internally by using a ferromagnetic metal; see Figure 2(a). In the latter case the effect is referred to as the anomalous Hall effect, since the external field is missing. However, chiral molecules covering the surface of a metal give rise to observations that are strikingly similar to the anomalous Hall effect.^17,18^ And without alluding to the possibility that the molecules somehow provide local magnetic moments, all ferromagnetically aligned, there is frankly no reason at all for these results.

**Figure 2:**
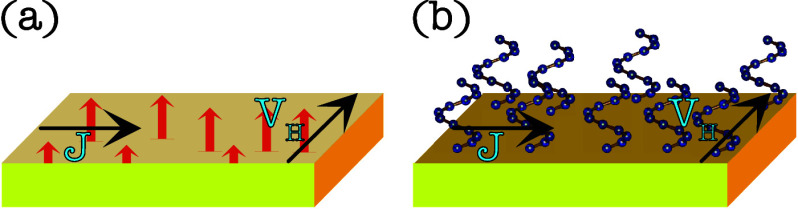
(a) Conventional setup for the anomalous Hall effect, in which a charge current *J* is applied to a ferromagnet resulting in a Hall voltage *V*_*H*_ perpendicular to both the current and magnetization. (b) Covering the surface of a nonmagnetic metal with chiral molecules leads to a similar effect.

In fact, these results appear to make theoretical physicists and chemists look the other way. As far as I am aware, no-one has addressed these particular problems deeply with the aim to provide a decent phenomenological explanation, let alone a comprehensive theory. Moreover, although the experimental results for good reasons may be interpreted as a version of the anomalous Hall effect, there is nothing cogently tangible in all this that does not allow for other interpretations. Might there be other reasons than magnetic for a charge current passing through a metal generating a voltage perpendicular to the current?

One proposal that has been pursued in the context of the chirality induced spin selectivity effect, and which has something to say about formation of local magnetic moments, is the spin interface, or spinterface for short.^22,23^ The proposal is that by interfacing a chiral molecule with a metal, a local magnetic moment emerges at or near the interface; see Figure 3. The model is based on the assumption that a spinterface is formed strictly in the interface region and is expected to be caused by exchange interactions between electrons in the molecule and metal.

**Figure 3:**
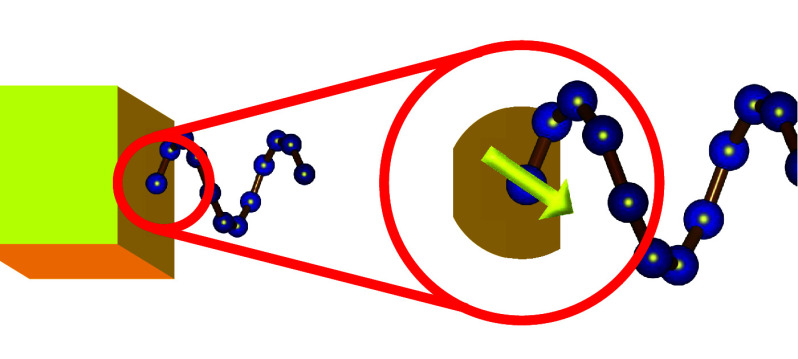
Spinterface formation between a chiral molecule adsorbed on a metal.

The emergence of spinterface have been reported for, e.g., Mn-phthalocyanine^24^ and zinc methyl phenalenyl,^25^ both adsorbed on ferromagnetic Co, and Alq_3_ (Al­(C_9_H_6_NO)_3_) interfaced with ferromagnetic Fe and Co.^26,27^ In all these examples, the spinterface is developed in the presence of a ferromagnet and despite being a challenge to manufacture set-ups where a spin-polarized density emerges in and around the molecular interface, it is not a complete surprise that a spin density is induced by proximity of the ferromagnet. A truly surprising spinterface was, by contrast, observed in C_60_/Al/Cu heterostructure,^28^ surprising since the constituents of the heterostructure are all diamagnetic.

While the phenomenology of the spinterface is appealing, there are few question marks that remain unanswered. The first is the perception that the chiral molecule is approximately a coil and, therefore, generates a magnetic field as response to the moving charges in the helical structure. Consequently a net flux of charges is necessary, something which is difficult to justify in equilibrium. This deficit was considered in ref 29, however, at the price of introducing a local spin splitting which is only phenomenologically justified. The local spin splitting is perceived to arise from the spin–orbit coupling. This assertion is simply not correct since, while the spin–orbit does induce an energy splitting between states, the states are nonetheless spin-degenerate. The splitting is not a Zeeman splitting. Second, the effect heavily relies on the interfaced metal having a strong spin–orbit coupling, which essentially rules out the possibility of obtaining the chirality induced spin selectivity effect using another substrate. However, the effect has been observed in setups with, e.g., graphene and Cu,^30,31^ both of which are not associated with strong spin–orbit coupling. Furthermore, the emergence of the spinterface in the C_60_/Al/Cu heterostructure^28^ suggests that the spin–orbit coupling of the substrate metal may be of subordinate importance. A third reason why the spinterface effect can be questioned is the necessity of a mean field acting within the system that facilitates formation of the local moment. And while a mean-field description may be sufficient in the context, in refs 22, 23, and 29 this mean field is introduced *ad hoc* and remains unspecified. Hence, in summary it is worth pointing out that while the spinterface should be considered phenomenologically sound, there are many open questions as to how to retain its features from microscopic modeling.

The only alternative theoretical model capturing the chirality induced spin selectivity effect based on spontaneous breaking of the time-reversal symmetry was introduced in two versions.^32,33^ The basic conceptual difference in comparison with previously launched theoretical contributions was inclusion of electron correlations, either through direct Coulomb interactions^32^ or indirectly via interactions between electrons and the moving nuclei.^33^ It should be remarked that the latter mechanism was independently proposed by others^34,35^ in the context of the chirality induced spin selectivity effect, however, not as the origin for local spin moment formation.

## Spontaneous Breaking of Time-Reversal Symmetry

Consider a molecular structure as a set of nuclei bonded to each other such that electron transfer between the nuclei is possible. The internuclei electron transfer is facilitated by both uncorrelated and correlated processes, where the former and latter refer to independent and interacting electrons, respectively. From a modeling perspective, the former is typically captured by any single particle, or quadratic, tight-binding model whereas the latter requires interactions of repulsive electron–electron or attractive electron–vibron type; see Figure 4. The term *vibron* can be regarded as the quantized nuclear or molecular vibrations in close analogy with phonon, albeit for finite structures. Although the microscopic details for inclusion of electron correlations is straightforward to capture in a Schrödinger equation, effectively these interactions can be formulated by operators like *∑*_*ij*_ *Un*_*i*_ *n*_*j*_ and *∑*_*ij*_ *J* **s**_*j*_·**s**_*j*_. Here, *n*_*i*_ and **s**_*i*_ denote the charge and spin operators, respectively, for electrons at *i*, and *U* and *J* are the associated Coulomb and exchange interaction parameters.

**Figure 4:**
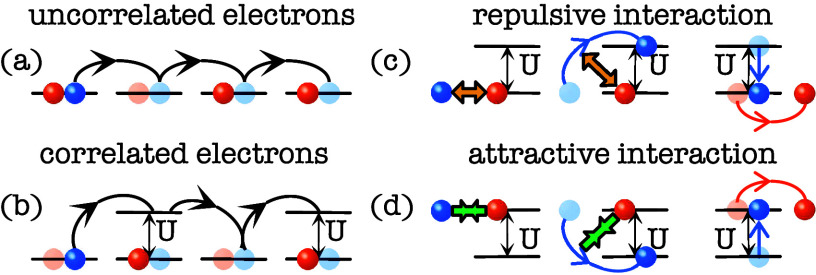
Uncorrelated and correlated electrons. (a) Uncorrelated electrons may occupy orbitals that are already occupied by an electron without the requirement of changing its energy. By Pauli exclusion, the two electrons have to have different spins, indicated with red and blue dots. (b) For correlated electrons such double occupancy has to be facilitated by an energy *U* corresponding to the repulsive or attractive interaction energy. (c), (d) Schematics over the processes involving double occupancy for (c) repulsive and (d) attractive interactions.

It should be recalled that any Coulomb interaction is accompanied by the spin exchange interaction which, for instance, leads to the Hartree–Fock approximation of interacting electrons. The electron–vibron interaction can be reformulated as a solely Fermionic interacting description where the Bosonic field of the nuclear vibrations mediates the interactions between electrons.

Regardless of the internal molecular interactions, time-reversal symmetry is maintained as long as the molecule can be considered isolated from its environment. Any inelastic process is inevitably accompanied by a compensating process such that the molecule remains a conservative entity.

The adsorbed molecule is quite a different object than the isolated one, not the least concerning its charge distribution which may be severely modified by the reservoir. In particular, electron correlations may facilitate strong such modifications; see Figure 5(a), where the site resolved charge distributions are calculated for helical (blue) *L*- and (red) *D*-enantiomers comprising 3 × 6 sites and mounted in the junction between nonmagnetic reservoirs. The corresponding charge distributions for the uncorrelated molecules are indicated by the cyan and magenta lines. These results demonstrate clearly that the electronic structure for uncorrelated structures barely changes upon interfacing with a macroscopic environment whereas for the correlated structures it leads to severe modifications throughout the molecule. Additionally, the correlated structures stabilize the spin configuration which is determined and protected by the molecular chirality; see Figure 5(b). The same setup for the uncorrelated molecule does not lead to an induced spin polarization, as would be expected from any noninteraction theory. Details about the calculations are presented in ref 21.

**Figure 5:**
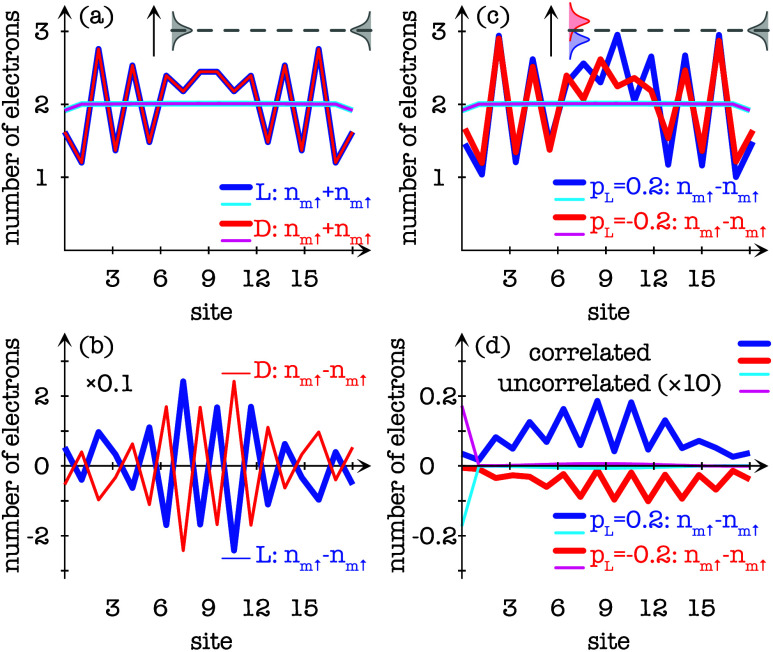
Site resolved charge and spin distributions of a 3 × 6-sites helix. (a) Charge distributions for (blue) *L*- and (red) *D*-enantiomers mounted in the junction between two nonmagnetic reservoirs and (b) corresponding spin polarizations. (c) Charge distributions for (blue) *L*- and (red) *D*-enantiomers mounted in the junction between a ferromagnetic and a nonmagnetic reservoir and (d) corresponding spin polarizations. In panels (a), (c), and (d) the corresponding results for uncorrelated electrons are shown by the cyan and magenta lines. Notice that the induced spin polarizations in the uncorrelated molecules in panel (d) are magnified by 10. Approach for calculations reported in ref 21.

The two different responses to interfacing with the reservoirs have deep consequences when one reservoir is replaced by a ferromagnet, see Figure 5(c),(d). While the uncorrelated molecules are barely affected by introducing a ferromagnet in the setup, the correlated molecule undergoes an additional substantial modification. First, it should be noticed that the charge distributions depends on the magnetization (parametrized in the vector **p**_*L*_, |**p**_*L*_| ≤ 1) of the ferromagnetic reservoir. As can be seen, the correlated molecule responds quite anisotropically to whether the magnetization **p** *_L_* = *p_L_* **ẑ** is positive (*p*_*L*_ > 0) or negative (*p*_*L*_ < 0). This anisotropic response, furthermore, influences the resulting molecular spin polarization such that these are not mirror images of one another. Instead, the ferromagnetic reservoir enhances the molecular spin polarization of one magnetization and diminishes it for the other. Such an anisotropic response is a clear effect of an intrinsic stable spin configuration, and for chiral molecules this spin configurations tends to have the character of a spin-density wave.

The origin of the chirality induced spin configuration is a confluence of chirality/spin–orbit coupling, electron correlations, and interfacing with the reservoir. Technically, it can be understood as the product of two imaginary contributions rendering a real quantity which acts as an effective magnetic field on the electrons. In a mathematical sense, the spin–orbit coupling is imaginary, albeit providing a Hermitian contribution to the Hamiltonian. The effect of the reservoir is an energy level broadening at the site adjacent to the reservoir; see the inset of Figure 5(a). This broadening, which contributes an imaginary part to the local spectrum, is the result of integrating out the macroscopic number of degrees of freedom associated with the reservoir. This process, hence, leads to converting the original Hermitian Hamiltonian model into a non-Hermitian. In the uncorrelated molecule, these two imaginary quantities, the spin–orbit coupling and level broadening, do not multiply and remain independent of one another. The electron correlations, by contrast, introduce processes in which those quantities do multiply, leading to a real valued field which acts like a spin splitting on the electronic structure. The typically associated processes are inelastic which open for leakage of, e.g., charge, energy, and linear and angular momentum to the reservoir. The leakage leads to stabilization of one specific spin singlet spin configurations whereas all others are damped out. The chirality of the molecule both determines and protects the favored spin configuration. There is no analogous mechanism in the uncorrelated molecule that can provide similar configurations.

## What May This Research Contribute to Science?

Knowing that chiral organic molecules acquire special spin configurations when interfaced with a reservoir may not have a huge technological impact. On the other hand, whether science should pertain to technological progression or not is not a question that we scientists are the best suited to address. Therefore, one should leave such questions aside to the benefit of putting an emphasis on the scientific questions themselves.

A reason why the formation of a steady state spin configuration is scientifically interesting is because it challenges the conventional theory for magnetism and symmetry considerations. The formation of a spin configuration is in itself not controversial; however, that it remains in this state for a long enough time to be measured by one or another means is controversial. First and foremost it challenges time-reversal symmetry. Second, spin configurations often come in pairs, so-called Kramers doublets and should one spin configuration be determined by chirality, where is its Kramers counterpart?

Regarding time-reversal symmetry, the simple answer to why it is broken is because of inelastic scattering and the leakage to the reservoir. This breaks the reversibility within the system, what is lost cannot be entirely restored by winding time backward. This is the context in which the damping processes are crucial. The molecular chirality appears to favor one of the spin singlet spin configurations whenever there is a chance to lose the ones that are not equally favored. The configurations that are not favored by chirality are damped out by the leakage to the reservoir, leaving the favored one protected since the structural chirality is not easily switched into its mirror image.

This leads to answering the second question, concerning the Kramers’ doublet. Essentially, Kramers’ theorem states that any eigenstate of a half-integer total spin particle in a time-reversal symmetric system, is accompanied by another eigenstate with the same energy related through time reversal. Hence, the spectrum of a half-integer spin particle is doubly degenerate. The conjecture that can be drawn from the theoretical studies of chiral molecules, summarized in, e.g., Figure 5, is that the Kramers doublet is constituted by the two enantiomers since their corresponding spin configurations are energetically degenerate and opposite to one another. It is true that the two enantiomers are not connected via time reversal, that is, one cannot obtain the *D*-enantiomer from the *L* by reversing time. However, it is not clear that one can actually separate temporal and spatial degrees of freedom in the conventional sense. On the other hand, if the molecule would display structural fluctuations that switch its conformation between the two enantiomers, its accompanying spin configuration would also fluctuate between two mutual opposites and, therefore, average to a trivial spin distribution. The time-reversal symmetry breaking can be thought of as being protected by the structural stability.

One may, of course, question the applicability of Kramers’ theorem in the current context, since closed shell molecules cannot be considered being half-integer spin systems. And even in the open shell arrangement assumed when interfaced with a reservoir, it is questionable whether the system acquires a half-integer state. Therefore, arguing against the protected spin configuration from the point of view of the necessity of a Kramers’ doublet can perhaps be dismissed by inapplicability of the required assumptions.

There are, nonetheless, implications with these results that may impact the way catalytic reactions should be perceived. In particular, the results pertain to the oxygen reduction reaction since it has been shown that the reaction rate can be controlled by the magnetic properties of the reservoir providing electrons to the reaction.^36–39^ The meaning of this statement is that a ferromagnetic reservoir may enhance the reaction rate compared with a nonmagnetic reservoir. Moreover, it has been demonstrated that the reaction rate is also enhanced in the presence of chiral molecules adsorbed on the electron reservoir.^13,14,40^

The result that the spin polarization of the electrons being transferred in the catalytic reactions makes a difference is definitely novel and potentially important in the context of, for instance, electrocatalysis. Provision of spin-polarized electrons via chiral molecules may have the beneficial side effect of opening up electrocatalytic technology based on organic molecules rather than on rare and expensive transitions metals. One should, however, be cautious in making such promises since there may be presently unknown reasons to why such technology may not ever be realized. Changes in the way we perceive the fundamental processes are inevitably important since this is where progression of our scientific endeavors begin.
